# Real Time Imaging and Dynamics of Hippocampal Zn^2+^ under Epileptic Condition Using a Ratiometric Fluorescent Probe

**DOI:** 10.1038/s41598-018-27029-5

**Published:** 2018-06-13

**Authors:** Hema Santhakumar, Resmi. V. Nair, Divya Susan Philips, Sachin J. Shenoy, Anoopkumar Thekkuveettil, Ayyappanpillai Ajayaghosh, Ramapurath. S. Jayasree

**Affiliations:** 1 Division of Biophotonics and Imaging, Bio Medical Technology Wing, Sree Chitra Tirunal Institute for Medical Sciences and Technology (SCTIMST), Trivandrum, 695012 Kerala India; 2 Photosciences and Photonics Group, Chemical Sciences and Technology Division, CSIR-National Institute for Interdisciplinary Science and Technology (CSIR-NIIST), Trivandrum, 695019 Kerala India; 3 Division of In Vivo Models and Testing, SCTIMST, Trivandrum, 695012 Kerala India; 4 Division of Molecular Medicine, SCTIMST, Trivandrum, 695012 Kerala India

## Abstract

Zinc, the essential trace element in human body exists either in the bound or free state, for both structural and functional roles. Insights on Zn^2+^ distribution and its dynamics are essential in view of the fact that Zn^2+^ dyshomeostasis is a risk factor for epileptic seizures, Alzheimer’s disease, depression, etc. Herein, a bipyridine bridged bispyrrole (BP) probe is used for ratiometric imaging and quantification of Zn^2+^ in hippocampal slices. The green fluorescence emission of BP shifts towards red in the presence of Zn^2+^. The probe is used to detect and quantify the exogenous and endogenous Zn^2+^ in glioma cells and hippocampal slices. The dynamics of chelatable zinc ions during epileptic condition is studied in the hippocampal neurons, *in vitro* wherein the translocation of Zn^2+^ from presynaptic to postsynaptic neuronal bodies is imaged and ratiometrically quantified. Raman mapping technique is used to confirm the dynamics of Zn^2+^ under epileptic condition. Finally, the Zn^2+^ distribution was imaged *in vivo* in epileptic rats and the total Zn^2+^ in rat brain was quantified. The results favour the use of BP as an excellent Zn^2+^ imaging probe in biological system to understand the zinc associated diseases and their management.

## Introduction

Zinc is a nutritionally essential trace element in the human body associated closely with proteins and enzymes. Although not all essential trace elements have known function in neural activity, several trace elements such as zinc, manganese and iron are transported into the brain for neural function^1^. Chelatable zinc constitutes about 10% of total zinc in the brain which is mostly accumulated in the synaptic vesicles of glutamatergic neurons throughout the brain, particularly in hippocampus^2,3^ and the rest, 90% exists as zinc metalloproteins^4^. The co-localization of Zn^2+^ with glutamate in presynaptic vesicles led to the hypothesis that vesicular Zn^2+^ may involve in synaptic neurotransmission^5^ and function as a neuromodulator of γ-amino butyric acid (GABA) and N-methyl-D-aspartate (NMDA) receptors in mammalian brain^4–7^. Small amount of Zn^2+^ also enters the postsynaptic neurons through NMDA, AMPA receptors and Ca^2+^ channels and regulate the activity of target proteins^8,9^. Though it is established that Zn^2+^ containing neuronal circuits are associated with episodic memory, behavior, emotion and cognitive-mnemonic operations, the exact role of vesicular Zn^2+^ remains obscure. Moreover, Zn^2+^ deficiency and dyshomeostasis is reported as a risk factor for epileptic seizures, Alzheimer’s disease, Parkinson’s disease, depression, and other neurodegenerative disorders^10,11^. Recent reports also suggested that the Zn^2+^ mobilization from presynaptic to postsynaptic neuron improves the social interaction in autism spectrum disorders^12^ and low nanomolar concentration of Zn^2+^ in Artificial cerebrospinal fluid is critical for synaptic activity^13^. In addition, cancer chemotherapy causes disruption of vesicular Zn^2+^ stores in hippocampal mossy fiber terminals which leads to decrease in hippocampal neurogenesis. A recent study reported that the dietary Zn^2+^ supplement can act as a simple alternative treatment for hippocampal neurogenesis to ameliorate Chemotherapy-induced cognitive impairment^14^. Hence, detection of Zn^2+^ concentration and its dynamics in both physiological and pathological conditions pose high demand. Better understanding on these factors is expected to throw more light on the functions and pathophysiology of some of these diseases and will be helpful in designing better strategies for their management. With these backgrounds, we tried to elucidate the dynamics of Zn^2+^ under epileptic condition, *in vitro* and *in vivo* using a Zn^2+^ specific fluorescent ratiometric probe.

Among the various techniques for the detection of metal ions, optical methods are well accepted due to high sensitivity, chemical inertness, wide dynamic range and reliable operation. Many of the optical techniques have already been well established for the detection of various metal ions *in vitro*, *in vivo* and in environmental conditions^15–21^. Among optical probes, fluorescence-based ion detection approaches/probes received significant attention because of its sensitivity, high spatial & temporal resolution, and possibility of non-invasive real-time detection in cells and in animals^22^. Fluorescent probes developed for biological Zn^2+^ detection uses a variety of mechanisms like internal charge transfer (ICT), photo-induced electron transfer (PET), excited state proton transfer, fluorescence resonance energy transfer (FRET) etc.^17,23–27^. PET mechanism commonly shows an increase or decrease in emission intensity rather than the spectral shift in either absorption or emission spectra. Hence compared to other fluorescence based mechanisms like PET, internal charge transfer is ideal as there is a spectral red-shift that leads to fluorescence color change for easy detection of metal ions. ICT occurs from an electron donor to an electron acceptor within fluorophores. In addition, ICT exhibits larger Stokes shift upon metal ion binding to the acceptor. Among them, red emitting fluorescent probes are in high demand because their spectral window lies outside the range of autofluorescence from biological specimens and are well suitable for the endogenous Zn^2+^ detection^28^. In addition, ratiometric fluorescent probes with two different measurable signals in the presence and absence of analytes are of great interest^29–31^. Ratiometric measurements not only increase the sensitivity of detection but also eliminate the artefacts caused by environmental factors such as variation in probe concentration, excitation intensity fluctuation, photobleaching, etc.^32^. Even though a few ratiometric fluorescent probes with increase/decrease in the intensity of red emission are available for endogenous Zn^2+^ detection *in vivo*^28^, no probes with dual functions like change in fluorescence and ratiometric quantification for Zn^2+^ detection are available thus far. Hence, fluorescent probes with these dual functions are highly demanding for visualization and accurate quantification^33^. We have designed few dual function fluorescent probes to detect biologically important analytes and pathological conditions^34–39^.

In the present study, a bipyridine bridged bispyrrole (BP) dual function probe is used for the detection and quantification of Zn^2+^ with visible light excitation, which may otherwise cause damage to living cells, is used. There has been little advanced evidence to portrait the dynamics/translocation and the pathway of released metal ion under various stimulating conditions^40^ and the modulation of NMDA/GABA receptors by the released Zn^2+^ for synaptic plasticity^41^. Moreover, little efforts have been made to the evaluation of the Zn^2+^ release *in vivo*. Herein, we describe the dynamics of Zn^2+^ in the hippocampal neurons under *in vitro* and *in vivo* epileptic conditions using a highly selective and sensitive fluorescent probe, BP.

## Results and Discussion

### Design and Optical response of BP towards Zn^2+^

**Figure 1:**
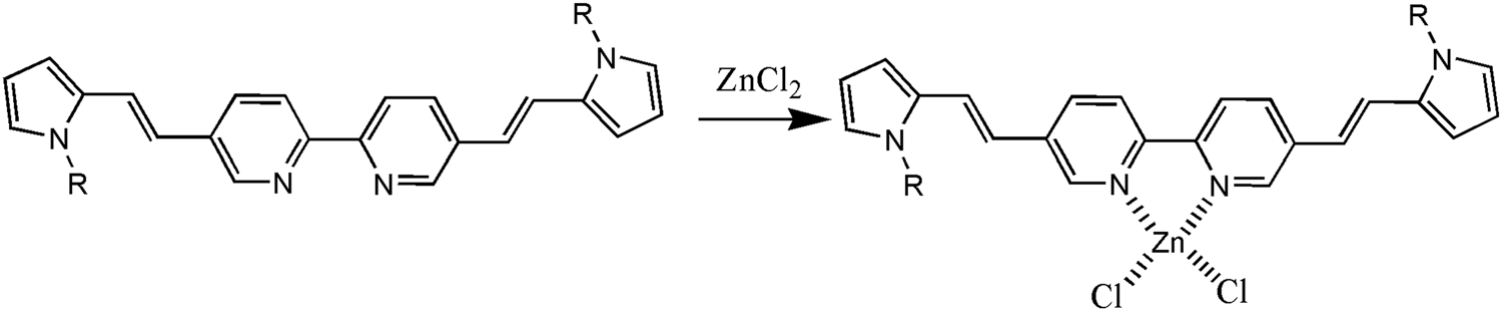
Structure of BP and the complex formation during zinc detection.

Molecular probes that respond to Zn^2+^ with a significant change in fluorescence emission are very attractive due to the importance of Zn^2+^ detection in neurobiology. The preparation and properties of three donor – π – acceptor - π- donor based molecular probes and its ratiometric fluorescence response towards Zn^2+^ was reported earlier^42^. Therein pyrrole (BP), thiophene (BT) and N-alkoxy aniline (BA) rings as donor moieties which were linked to a bipyridine binding site have been used. It was observed that BA was not a suitable Zn^2+^ detection probe as it quenches the emission in the presence of a variety of cations including Zn^2+^ whereas BP and BT showed excellent fluorescent ratiometric zinc ion detection potential. We have chosen BP for the current study as it gives an emission shift from green to red (with Zn^2+^) which is preferred for biological and animal imaging due to the deep tissue imaging potential of NIR wavelengths and the less interference of autofluorescence in this region. Here, the probe was designed to contain a 2,2′-bipyridine as acceptor moiety (metal chelating centre) for selective binding of Zn^2+^, which is connected to pyrrole donor groups through a π-bridge (Fig. 1). The binding of cationic Zn^2+^ to the bipyridine moiety generates a partial positive charge, which can be transferred to the donor moiety upon excitation since the π-bridge connects the two moieties. In addition, changing from a non-planar to planar conformation on Zn^2+^ binding enhances the intramolecular charge transfer in the probe. Hence, the probe exhibits a strong intramolecular charge transfer upon excitation, resulting in a change in both the excitation and emission spectrum on Zn^2+^ binding. The binding of Zn^2+^ induces a decrease in HOMO-LUMO energy gap which is attributed to the observed red shift in the emission of the probe upon complexation. The binding constant of Zn^2+^ complexation with BP probe was calculated as 3.6 × 10^5^ M^−1^. The Zn^2+^ binding to BP probe is confirmed with Surface Enhanced Raman Scattering (SERS) and Fourier Transform Infrared (FTIR) spectroscopic techniques. SERS spectra of both BP and BP + Zn^2+^ showed well defined peaks with a clear metal – nitrogen vibration peak in the 638–684 cm^−1^ region (Fig. S1). FTIR spectrum showed a metal-nitrogen stretching at 450 cm^−1^ corresponding to the binding of Zn^2+^ to the nitrogen group of the bipyridine acceptor moiety (Fig. S2).

**Figure 2:**
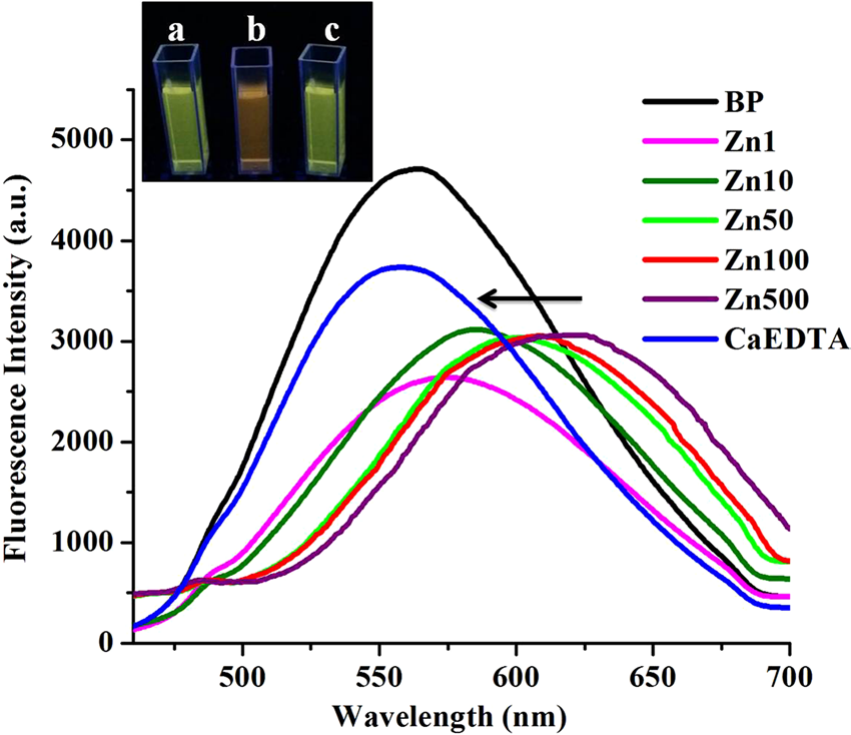
Fluorescence spectra of BP (6 µM) with ZnCl_2_ at different concentrations (1,10,50,100,500 µM) in PFS. Arrow indicates the reversal of fluorescence shift on addition with CaEDTA. Inset shows the visual fluorescence color change of BP (**a**), after the addition of ZnCl_2_ (**b**) and after the addition of CaEDTA (**c**).

The optical properties of BP were evaluated in physiological condition before proceeding with the biological applications. Remarkable difference in the optical behaviour was observed in physiological condition, compared to that in the organic buffer^42^. In Phosphate Free Saline buffer (PFS, pH 7.4), it has an absorption in the visible region at 410 nm, which shifts to 421 nm, upon addition of Zn^2+^ (Fig. S3). The emission profile of BP (6 µM) also shows a concentration dependent red shift on addition of Zn^2+^, from the original position of 565 nm (Fig. 2). Addition of a Zn^2+^ chelator, CaEDTA (50 µM) reverts the emission peak to the original position of 565 nm (Fig. 2), due to the de-complexing of Zn^2+^ from BP. This confirms that the observed red shift in the emission of BP is a consequence of Zn^2+^ binding. The probe has a large stoke shift of 155 nm in PFS, which is highly favourable for biological imaging to reduce the interference from spectral overlap and quenching.

### *In vitro* cellular cytotoxicity and imaging of exogenous Zn^2+^ in C6 glioma cells

**Figure 3:**
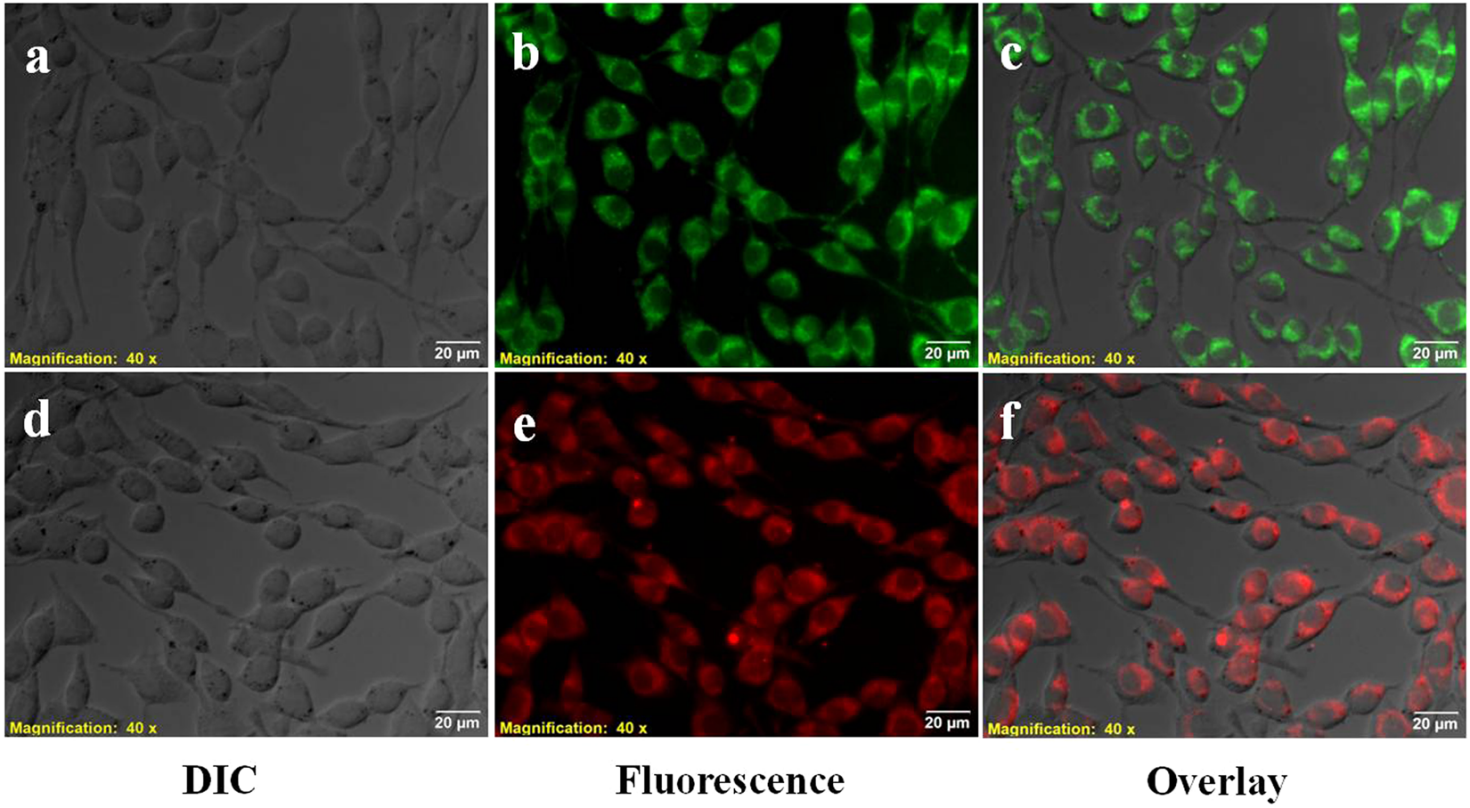
Fluorescence microscopic images of Zn^2+^ detection in C6 Glioma cells using BP. DIC, fluorescence and overlay images of cells incubated with BP alone (**a**,**b**,**c**) and cells pre- treated with Zn^2+^ and then incubated with BP (**d**,**e**,**f**). Magnification 40 × and scale bar = 20 µm.

On proving the Zn^2+^ detection capability of BP in PFS, the suitability of the probe for live cell imaging was assessed by long-term cellular cytotoxicity study in C6 rat brain glioma cells using standard MTT assay. A concentration of 6.25 µM of BP was found to be non-toxic with more than 85% and 70% cell viability for 24 and 48 h incubation period, respectively (Fig. S4). Since 6 µM of BP was found to be non-toxic over a period of 48 h, this concentration is considered as safe for Zn^2+^ detection under complex physiological condition that require prolonged probe treatment. The internalization of BP was proven in C6 glioma cells with the observed good intracellular distribution (Fig. 3b). To investigate the *in vitro* Zn^2+^ detection potential of BP, Zn^2+^ (50 µM) was exogenously added and efficiently transported into the cells using a Zn^2+^ specific ionophore, pyrithione (2-mercaptopyridine N-oxide). Such ionophores are extensively used in experimental biology, which can act as passive shuttle for ions to move across cell membranes^43^. Addition of BP to the Zn^2+^-pyrithione complex loaded cells gave intense red fluorescence as indicative of the Zn^2+^ detection by BP, whereas cells incubated with BP alone showed green fluorescence (Fig. 3b,e).

### Imaging the endogenous Zn^2+^ in acute hippocampal slices

**Figure 4:**
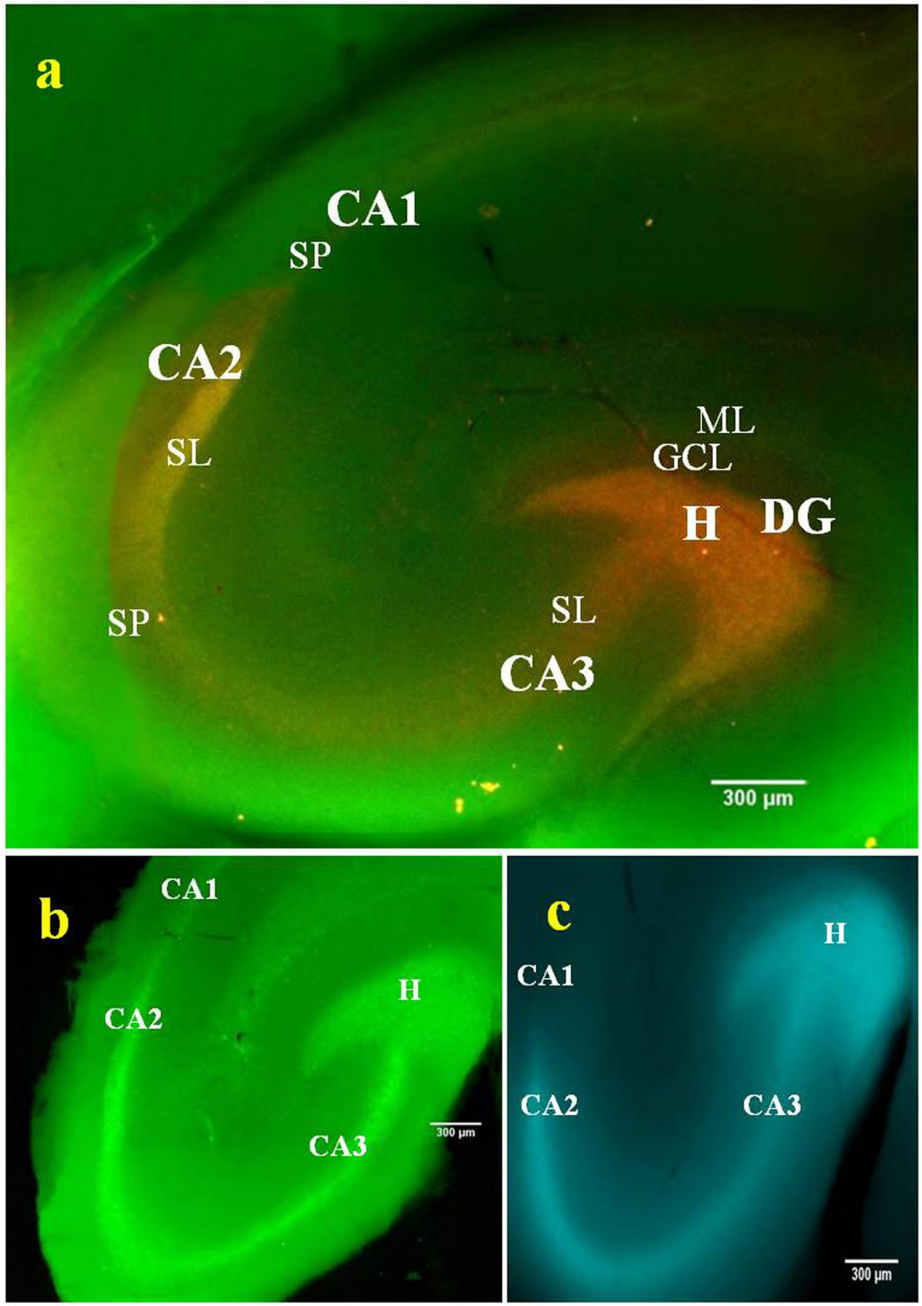
Fluorescence imaging of endogenous Zn^2+^ in acute rat hippocampal slices. Slice incubated with BP & Fluozin-3 AM and imaged using 460–490 nm excitation and 520 nm emission respectively (**a** & **b**). Slice incubated with TSQ and imaged using 330–385 nm excitation and 420 nm emission (**c**). H marks the area of dentate Hilus, DG- Dentate gyrus, CA1, CA2 & CA3 – Cornu ammonis 1, 2 & 3, SL- Stratum lucidum, SP- Stratum pyramidale, GCL- Granular cell layer, ML- Molecular layer. 4 × Magnification, images stitched manually, scale bar = 300 µm.

Encouraged by the results of externally supplied Zn^2+^ imaging in glial cells, BP was further tested for its efficacy to image the endogenous Zn^2+^. For this purpose, hippocampal slices of 400 µm thickness were prepared from adult Sprague-Dawley rats (Fig. S5). BP incubated brain slices showed maximum Zn^2+^ induced red fluorescence in the hilar region of dentate gyrus (H) as well as in the stratum lucidum (SL) of CA2 and CA3 (Fig. 4a) confirming the presence of high concentration of Zn^2+^ containing synaptic vesicles in these two regions^44^. This result also confirms the efficacy of BP to detect vesicular Zn^2+^ in neurons. The result was validated using standard cell-permeable dyes Fluozin-3 AM and TSQ. These are Zn^2+^ sensors that show characteristic increase in green and blue fluorescence intensity respectively (Fig. 4b,c) on binding with the endogenous Zn^2+^. A visible fluorescence shift from green to red shown by BP is much appreciated for imaging of Zn^2+^ when compared to the intensity change by the standard probes. Further, to check whether BP detects vesicular or extracellular Zn^2+^, a membrane impermeant chelator CaEDTA and membrane permeant chelator TPEN were added separately to different sets of hippocampal slices incubated with BP. Addition of these chelators caused an immediate depletion of red fluorescence from all regions that have Zn^2+^ containing innervations, which include hilus of the dentate gyrus, mossy fiber terminals of CA3, radiatum and oriens of CA1 (Fig. S6). The decrease in Zn^2+^ induced red fluorescence of BP upon addition of TPEN was higher than CaEDTA (Fig. S6 Inset), which indicates that BP is efficient in detecting vesicular Zn^2+^.

### Imaging the dynamics of endogenous Zn^2+^ in hippocampal slice under epileptic condition

**Figure 5:**
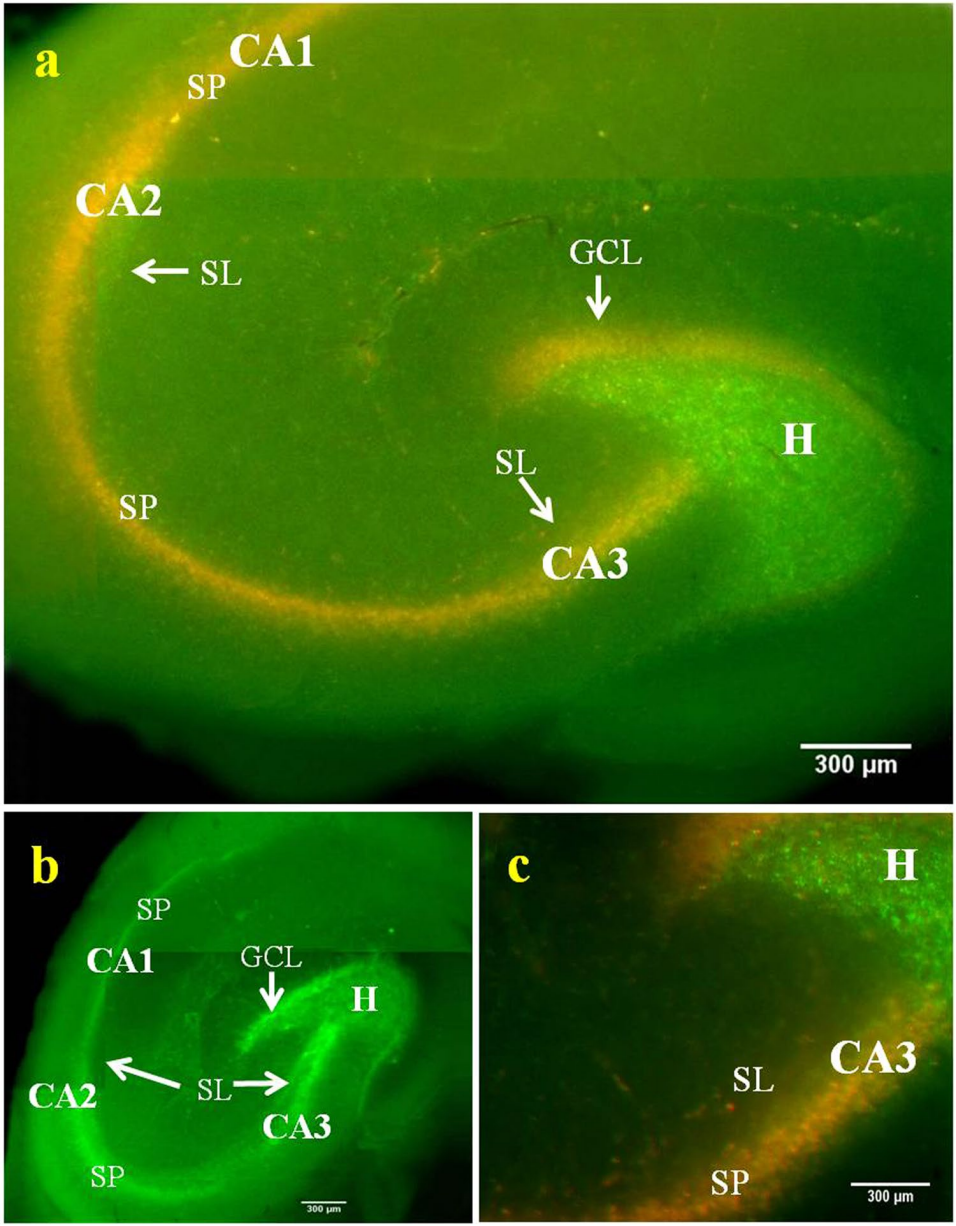
Dynamics of synaptically released Zn^2+^. Translocated Zn^2+^ is imaged using BP (6 µM) under elevated K^+^ stimulation. The red fluorescence from the H of DG and SL of CA3 disappears completely and gets shifted towards GCL of DG, SP of CA3 and CA1 pyramidal body layer compared to unstimulated slice of Fig. 4a. (**c**) shows the higher magnification (10×) image of (**a**) showing clear translocation of Zn^2+^ as imaged using BP. Fluozin-3 AM also shows similar pattern of Zn^2+^ translocation like BP, but with less sensitivity as seen in (**b**).

In order to study the dynamics of Zn^2+^ during a disease condition, BP was further used in a clinically relevant situation like epilepsy. It is reported that Zn^2+^ stored in presynaptic vesicles are released into the synaptic cleft similar to neurotransmitters upon depolarization of neuronal membrane and the release is higher under pathological conditions, similar to epilepsy^1,45^. Hence, studying the dynamics of Zn^2+^ under epileptic condition is important to understand the etiology and manifestation of epilepsy. Here, the induction of synaptic release of Zn^2+^ in hippocampus to mimic epileptic condition was done by exogenous K^+^ stimulation. Potassium stimulation is one of the common techniques to induce *in vitro* epileptiform activity^46,47^. The nerve terminals in the hippocampus were stimulated to depolarize with the addition of extracellular potassium ions. On extracellular K^+^ stimulation, a dramatic reduction in the Zn^2+^ induced red fluorescence was observed in the hilus, stratum lucidum of CA3 and CA2 (Fig. 5a), compared to that of the unstimulated condition (Fig. 4a). Reduction in the red emission suggests that during K^+^ stimulation, Zn^2+^ is released into the synapse from presynaptic neuronal cells. This study gives a visual picture of the presence of highly concentrated histochemically active zinc ions in the synaptic vesicles of mossy fiber boutons and the synaptic release of it under stimulation^48,49^. Additionally, an interesting phenomenon of the Zn^2+^ translocation was observed during epileptic stimulation. The translocation of Zn^2+^ from the hilus to the GCL of DG and from stratum lucidum to the stratum pyramidale of CA3 & CA2 was observed. The translocated Zn^2+^ was also visible in the postsynaptic pyramidal neurons of CA1 (Fig. 5a). CA1 has relatively low levels of Zn^2+^ than CA2 in stimulated epileptic slice (Fig. 5a), while it is almost absent in unstimulated control slice (Fig. 4a). Based on these findings, we have mapped a connectome for the Zn^2+^ translocation in hippocampus during epilepsy as DG- > CA3- > CA2- > CA1. Imaging of the translocation of Zn^2+^ in whole hippocampal slices has not been demonstrated so far, though there are a few studies that reports the translocation in single neuronal cells^49,50^. Fluozin-3 AM also showed similar translocation pattern (Fig. 5b). However the detection was not efficient as that of BP. The translocation exhibited by Zn^2+^ pose a need for re-evaluation of the conventional concept of synaptically released neurotransmitters, which normally would bind to the receptors in the postsynaptic neuronal cells to activate/inhibit the membrane channels for signal transduction. The entry of Zn^2+^ into the postsynaptic cytosol would allow it to interact with many cytosolic macromolecules, unlike the conventional neurotransmitters^51^. This unique feature of vesicular Zn^2+^ is responsible for the increase in Zn^2+^ levels within the post-synaptic cytosol causing neurodegenerative diseases, like epilepsy^52^. Some of the previous findings suggested that in physiological conditions, the function of vesicular Zn^2+^ after release in the synaptic junction is to bind to the NMDA and glycine receptors for modulating their function and also to play a role in synaptic plasticity^53^. However, the function of vesicular Zn^2+^ after synaptic release and after translocation in postsynaptic cytosol remains largely unclear under pathological conditions like epilepsy. Hence the development of probes like BP would largely help to visualize and study the dynamics of Zn^2+^ under neurodegenerative disorders.

### Detection of Zn^2+^ and its dynamics in hippocampal slices using Raman spectroscopy

To confirm the results, the molecular fingerprinting of the hippocampal slices along with BP in normal and epileptic condition was studied by recording Raman spectra from the hilar region. All slices showed characteristic vibrational peaks of phospholipids, aliphatic amines, tryptophan, tyrosine etc. of hippocampal slices (Fig. S7). The vibrational peak at 678 cm^−1^ corresponding to metal–nitrogen bond formed between BP and Zn^2+^ in the hilar region is prominent in case of unstimulated slice. Whereas, the intensity of this peak is decreased in the case of stimulated epileptic slice, indicating the release of Zn^2+^ from the hilar region. It is in good agreement with the fluorescence imaging and SERS spectral data of the Zn^2+^-BP binding study. Complete Raman peak assignments in control and stimulated slices are available (Table S1).

### Ratiometric imaging

#### Ratiometric imaging and quantification of exogenous Zn^2+^ in C6 glioma cells

**Figure 6:**
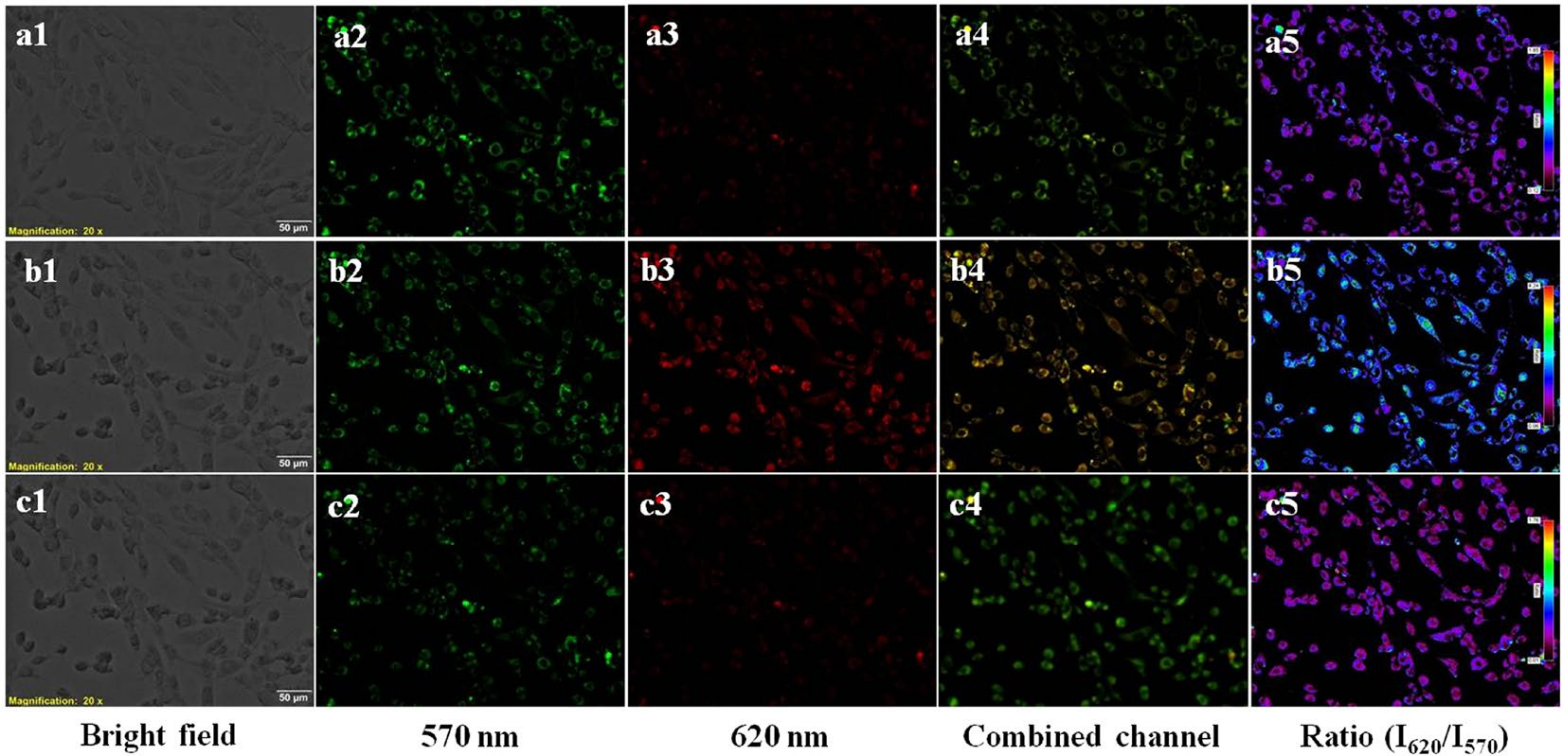
Ratiometric time lapse imaging of C6 Glioma cells for Zn^2+^ detection, with BP. Cells incubated with BP for 30 min (a1–a5). BP incubated cells treated with Zn^2+^-pyrithione complex for 15 min (b1–b5) and treated with TPEN for 20 min (c1–c5). Magnification 20×, scale bar = 50 µm. Ratio bar from 0.12 to 1.85 in a5, 0.06 to 4.24 in b5, 0.01 to 1.76 in c5.

Further, to prove the ratiometric imaging potential of BP, a live cell imaging of C6 glioma cells treated with BP, Zn^2+^ and Zn^2+^ chelator TPEN was performed using excitation filters in the range 470–495 nm. The first 30 min incubation of BP showed high fluorescence intensity in the green channel (Fig 6,a2). Another 15 min incubation of Zn^2+^ led to a marked elevation of fluorescence intensity in the red channel (Fig. 6,b3), accompanied by the reduced fluorescence intensity in the green channel (Fig. 6,b2). With another 20 min incubation with the membrane permeable metal ion chelator TPEN, a decrease in fluorescence in the red channel (Fig. 6,c3) and an increase in fluorescence in the green channel (Fig. 6,c2) was observed, due to the reversible Zn^2+^ chelation effect. In all cases, ratiometric images were generated based on the ratio of intensity at red and green channels, i.e.I_620_/I_570_ (Fig. 6,a5–c5). The average ratio of emission intensities after each treatment was calculated and the increment in emission intensity ratio from 0.48 in case of BP to 2.86 on addition of Zn^2+^ was observed (Fig. S8). This confirms that the enhancement of signal in the red channel is due to the coordination of Zn^2+^ with BP, ruling out the influence of factors like autofluorescence, proton flux into the cells, light scattering or probe photo activation due to over exposure. The finding qualifies the candidature of BP in the real time monitoring of intracellular Zn^2+^ concentration under biological conditions.

#### Ratiometric imaging of the dynamics of endogenous Zn^2+^ in acute hippocampal slice under epileptic condition

**Figure 7:**
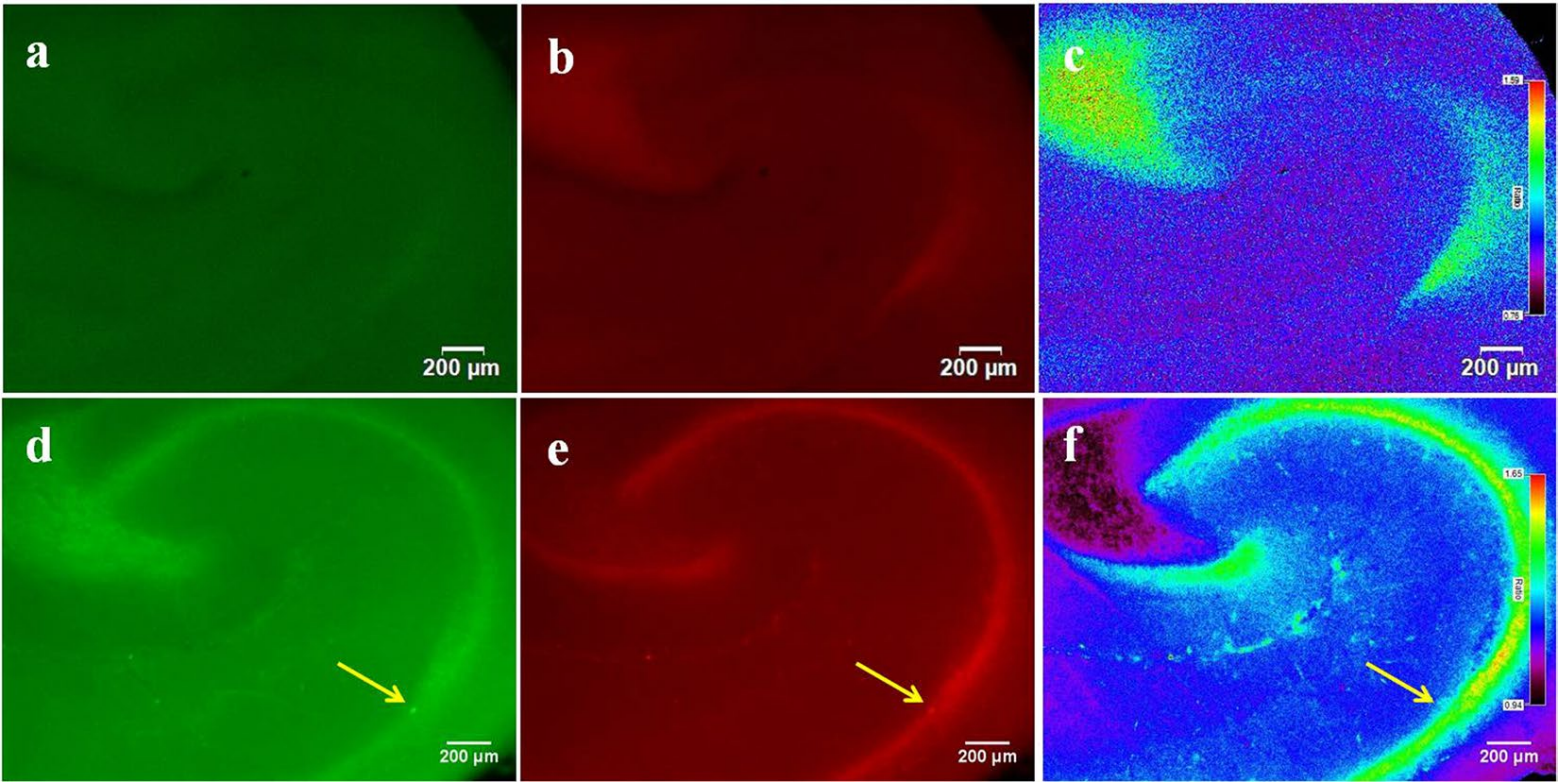
Ratiometric fluorescence imaging of endogenous Zn^2+^ and its dynamics showing the translocation. Control slice with BP and stimulated epileptic slice with BP are shown in the 1^st^ and 2^nd^ rows respectively. (**a**,**d**) represents fluorescence images with emission collected at 510–550 nm and (**b**,**e**) represents that collected at 575–625 nm. Ratiometric images (I_620_/I_570_) generated from 1^st^ and 2^nd^ columns are shown in c and f. Magnification 1.25 × (control), 4 × (stimulated), scale bar = 200 µm. Ratio bar represents Zn^2+^ concentration from low (black) to high (red). Arrows in (**d**,**e**) indicates the intense fluorescence due to artefact which is cancelled out in (**f**).

Further, the endogenous Zn^2+^ and its dynamics in hippocampal slices were also measured ratiometrically under normal and epileptic condition. The ratiometric image generated from the green and red channel images provide an indication about the quantity of Zn^2+^ in each area of hippocampal slices (Fig. 7). The average ratio of emission intensities from different areas of hippocampal slices were also calculated (Fig. S8). The emission intensity ratio was found to decrease in the hilus and SL of K^+^ stimulated epileptic slice compared to that of the control slices. At the same time, K^+^ stimulated slice showed higher ratio in the GCL of DG, CA3 pyramidal layer and in the CA1 pyramidal body, where the ratio was very low in control slice. This confirms the translocation of endogenous Zn^2+^ in vesicles from presynaptic to postsynaptic cells under stimulated epileptic condition. The fluorescence intensity in the hippocampal slices could be severely influenced by factors like non-uniformity of the slice thickness, excitation intensity variation and/or artefacts associated with probe concentration. However, ratiometric measurement is expected to overcome such problems, because the ratio between the two fluorescence intensities is independent of such factors. For example, the intense fluorescence at the end of CA3 in Fig. 7d,e due to artefacts was cancelled out in the ratiometric image of Fig. 7f. The concentration of vesicular Zn^2+^ and its translocation to the postsynaptic cytosol under pathological conditions like epilepsy continue to be a challenge for neurobiologists. Hence, the development of ratiometric probes like BP would solve these challenges to understand the concentration of Zn^2+^ translocation under neurodegenerative conditions in greater depth for successive treatment.

#### Quantification of total Zn^2+^ in rat brain

Following the detection and ratiometric quantification of translocated Zn^2+^ in brain slices using fluorescence microscopy, the amount of free Zn^2+^ in the whole brain was quantified by measuring the pixel intensity ratio of Zn^2+^ in brain sections using an *in vivo* optical imaging system (Fig. S9). The total amount of free Zn^2+^ estimated likewise from pixel intensity of brain section/pixel intensity of BP alone was of the order of ~0.4356 M (~2.85 mg) for a brain weighing 2.4 g.

#### *In vivo* imaging of brain Zn^2+^ under epileptic condition

Finally, the efficacy of BP to image the Zn^2+^ release *in vivo* during epileptic condition was tried on pilocarpine (350 mg/kg) induced epileptic animal models. Development of epileptic model and attainment of status epilepticus were ascertained by assessing the behavioural changes (see supplementary information) of the animals through continuous videography after the injection of pilocarpine. For imaging, the animals were anesthetized and perfused with 80 µM of BP in PFS through external carotid artery, after 24 h of attaining status epilepticus. As the presence of blood brain barrier (BBB) is expected to prevent the entry of BP into the brain, prior to BP infusion BBB was disrupted chemically using intra carotid artery infusion of mannitol to ensure the access of BP to the brain. During *in vivo* imaging, it is made sure that all rats were stable with normal respiration, and no motion artefacts due to seizures interfered. The animals were imaged at 430 nm excitation and 620 nm emission. An intense fluorescence from concentrated Zn^2+^ present in hippocampus was visible in the left hemisphere of brain in BP infused rats (Fig. S10). The fluorescence enhancement was expected in the left hemisphere, because intra carotid artery infusion of BP was done through left external carotid artery. No intense fluorescence signal was observed on the right hemisphere and also in the case of PFS treated rats. The results are in support of the proof of the potential of BP for real time imaging of Zn^2+^ in epileptic and other neurodegenerative conditions, *in vivo*.

To conclude, imaging of Zn^2+^ in the *in vitro* and *in vivo* conditions was successfully performed using the ratiometric bispyrrole probe (BP). Ratiometric imaging with BP could clearly visualize and measure the exogenous Zn^2+^ in cells and endogenous Zn^2+^ in hippocampal slices without the interference from artefacts. Moreover, a visual picturization of Zn^2+^ dynamics in hippocampus during *in vitro* epileptic condition could be mapped using this probe. Results of the present study indicate that the release and translocation of high concentration of Zn^2+^ from the presynaptic terminals is involved in the pathogenesis of neurodegenerative disease like epilepsy. We expect that the real time imaging and quantification of Zn^2+^ in pathophysiological conditions will open up new arena in the field of brain research and Zn^2+^ biology. Strategies to modify the probe to enable brain specificity and easy entry into the brain will facilitate in-depth understanding of neurodegenerative diseases like epilepsy.

## Materials and Methods

### Reagents

Reagents, chemicals and media were obtained from commercial suppliers and used without further purification: FluoZin-3 AM and TSQ (N-(6-Methoxy-8-Quinolyl)-p-Toluenesulfonamide) were purchased from Molecular Probes, Invitrogen. Ethylenediaminetetraaceticacid disodium calcium salt (CaEDTA), N,N,N′,N′-tetrakis(2-pyridylmethyl)ethylenediamine (TPEN) and 2-mercaptopyridine N-oxide (pyrithione) were obtained from Sigma-Aldrich. All other chemicals and media like Dulbecco’s Modified Eagle’s Medium/Nutrient mixture F12 Ham (DMEM/F12, 1:1), FBS, Penicillin, Streptomycin and Amphotericin B were purchased from HiMedia; C6 Glioma cells from ATCC.

### Imaging System and Instrumentation

The imaging system comprises of an inverted fluorescence microscope (IX51; Olympus, Tokyo, Japan), Rolera-XR Mono Fast 1394 Cooled digital camera (QImaging) and NIS Elements Advanced Research software (Nikon Instruments Inc.) for image acquisition. The microscope was equipped with a mercury arc lamp, a 4X/0.13 objective lens and mirror unit U-MWB2 WB (Excitation/Emission filter 460–490/520) was used for imaging hippocampal slices. Vibroslice NVSL with manually advanced tissue bath was used for brain slicing. For ratiometric imaging, we used an inverted fluorescence microscope (IX83; Olympus Corp., Tokyo, Japan) with a cooled CCD camera (XM10, monochrome, Olympus). The microscope was equipped with a metal halide lamp (X-Cite, series 120PC Q). An objective lens, LUCPLFLN 40X PH/0.6, Olympus for cell imaging and PlanApoN 1.25X/0.04, Olympus, for hippocampal slices were used with an excitation filter (470–495, Olympus), a dichroic mirror (DM505, Olympus), and two emission filters (510–550, 575–625, Olympus). The ratiometric imaging system was controlled with CellSens Imaging software (Olympus). All spectroscopic measurements were conducted at RT. Absorption, emission and IR spectra were recorded with UV-1800 Shimadzu UV-Vis spectrophotometer, FP-8200 Jasco spectrofluorometer and Fourier transform infrared spectrometer Carry 600 (Agilent Technologies) respectively. IR Spectra were recorded in the range of 400 to 4000 cm^−1^ in transmission mode using KBr pellet method spanning over 32 scans. Raman spectra of the slices and the material were investigated with the spectral Imaging Mode of the confocal Raman Microscope (alpha300R, WITec Inc. Germany). *In vivo* animal imaging and the *ex vivo* imaging for zinc quantification in brain was done with a live animal optical imaging system (Xenogen, IVIS Spectrum).

### Synthesis of BP

For the preparation of the ratiometric probe BP, sodium hydride (12 mmol) was added slowly to a solution of 2,2′-bipyridyldiphosphonate (2 mmol) and N-alkylpyrrole-2- carboxaldehyde (4 mmol) in THF, and refluxed it for 12 h. Then the fluorescent reaction mixture was cooled and THF was removed under reduced pressure, which gives a pasty residue. The residue was suspended in water and extracted with dichloromethane. Brine was used to wash the organic layer and then it was dried over Na_2_SO_4_ to give the crude product. The product was further purified over silica gel (100–200 Mesh) by column chromatography using petroleum ether/ethyl acetate mixture as eluent. The methods in detail are reported previously^39^.

### *In vitro* cellular imaging of Zn^2+^

In order to study the *in vitro* imaging capability of BP and detection of Zn^2+^, C6 glioma cells incubated with 6 µM of BP for 30 min was used as controls. Further, BP was incubated with another set of C6 glioma cells pre-incubated with 50 µM of Zn^2+^ for 15 min along with 25 µM of 2-mercaptopyridine N-oxide (pyrithione), a Zn^2+^ selective ionophore. Imaging was performed using 460–490 nm excitation for both the groups, and 510–550 nm and 575–625 nm emission filters was used respectively for control and test group.

### Zn^2+^ detection in acute hippocampal slices

Acute hippocampal slices were prepared as described in the supplementary information. Slices were incubated with 6 µM of BP for 30 min at room temperature in PFS, continuously stirred with a jet of 95% O_2_ and 5% CO_2_. Cell-permeable form of dyes Fluozin-3 AM and TSQ (N-(6-Methoxy-8-Quinolyl)-p-Toluenesulfonamide) were used as positive controls for vesicular Zn^2+^ detection. Fluozin-3 AM (Kd (Zn^2+^) ~15 nM) is suitable for detection of Zn^2+^ concentrations in the 1–100 nM range. Fluozin-3 AM was incubated at a concentration of 2.5 µM in PFS for 15 min. TSQ is also selective for zinc in the presence of physiological concentrations of Ca^2+^ and Mg^2+^, and was incubated at a concentration of 90 µM for 20 min. The stained slices were washed with PFS to remove excess dye and observed under Olympus IX51 inverted fluorescence microscope at an excitation/emission wavelength of 460–490/520 nm for BP, Fluozin-3 AM and 330–385/420 nm for TSQ respectively. Fluozin-3 AM stained slices were imaged only after 30 min to allow the AM ester to cleave inside the cells for fluorescence emission.

### Induction of epileptic condition *in vitro*

The induction of epileptic condition in hippocampal slices was achieved by potassium chloride stimulation. Slices were loaded with 50 mM KCl for 15 min and incubated with BP at a concentration of 6 µM for 30 min before imaging.

### Ratiometric live cell imaging

During ratiometric imaging, cells were kept on the microscopic stage at room temperature. Addition of BP (6 µM), zinc (50 µM) as pyrithione complex or TPEN (50 µM) to the cells was performed directly on the microscopic stage by bath application to the media. Exposure time of excitation was kept constant until the completion of image acquisition. Ratiometric images were generated from the ratio of red channel image (575–625 nm) with the related green channel image (510–550 nm) with single excitation (470–495 nm) using Olympus software (CellSens). Furthermore, the average ratio of emission intensities F_575–625_/F_510–550_ upon excitation at 470–495 nm was calculated from 5 ROIs of different cells after every treatment. Also, examined the ratiometric measurement of endogenous Zn^2+^ and its dynamics in the hippocampal slices under KCl stimulion, using same excitation and emission filters. The average ratio of emission intensities F_575–625_/F_510–550_ was also calculated from 5 ROIs of different areas in the hippocampal slices under normal and stimulated epileptic conditions.

### *In vivo* imaging of Zn^2+^

All the animal experiments were carried out according to the principles set forth by the Committee for the Purpose of Control And Supervision of Experiments on Animals (CPCSEA) and were approved by the institutional animal ethics committee of Sree Chitra Tirunal Institute for Medical Sciences and Technology (B2982011 VIII). Adult female Sprague-Dawley rats weighing 150 g injected with saline were used as control (n = 3). Epilepsy was induced in animals of same weight (n = 6). Detailed methodology on the development of epileptic animal model is available in supplementary information. BBB was disturbed in both the control and epileptic rats with intra-carotid artery infusion of mannitol (see supplementary information) and the epileptic rats were perfused with 80 µM of BP in PFS into the external carotid artery and imaged with live animal imaging system (Xenogen, IVIS Spectrum). Control rats were perfused with PFS.

### Data availability

The datasets generated during and/or analysed during the current study are available from the corresponding author on reasonable request.

## Electronic supplementary material

Supplementary information
