# Supplementary material for: Real Time Imaging and Dynamics of Hippocampal Zn2+ under Epileptic Condition Using a Ratiometric Fluorescent Probe
Source: Sci Rep. 2018 Jun 13;8:9069. doi: 10.1038/s41598-018-27029-5 (PMC5998144; doi:10.1038/s41598-018-27029-5)
Supplement: Supplementary file 1 — Supplementary information [file 41598_2018_27029_MOESM1_ESM.pdf]

Supplementary Information for

Real Time Imaging and Dynamics of Hippocampal Zn^{2+} under Epileptic Condition Using a Ratiometric Fluorescent Probe

Hema Santhakumar[a], Resmi. V. Nair[a], Divya Susan Philips[b], Sachin J. Shenoy[c], Anoopkumar Thekkuveettil[d], Ayyappanpillai Ajayaghosh[b] and Ramapurath. S. Jayasree[a*]*

[a]Division of Biophotonics and Imaging, Bio Medical Technology Wing, Sree Chitra Tirunal Institute for Medical Sciences and Technology (SCTIMST), Trivandrum 695012, Kerala, India.

[b]Photosciences and Photonics Group, Chemical Sciences and Technology Division, CSIR-National Institute for Interdisciplinary Science and Technology (CSIR-NIIST), Trivandrum 695019, Kerala, India.

[c]Division of In Vivo Models and Testing, SCTIMST, Trivandrum 695012, Kerala, India.

[d]Division of Molecular Medicine, SCTIMST, Trivandrum 695012, Kerala, India.

*Corresponding authors at: Sree Chitra Tirunal Institute for Medical Sciences and Technology, Tel.: +91 471 2520273; +91 9495948221. Email address: jayasree@sctimst.ac.in and CSIR-National Institute for Interdisciplinary Science and Technology, Tel.: +91 471 2490324; +91 471 2493599, 9446059059. Email address: ajayaghosh@niist.res.in

EXPERIMENTAL SECTION

In vitro studies:

Cell Culture

C6 glioma cells (rat glial tumor cell line) were routinely cultured in Dulbecco's Modified Eagle's Medium/Nutrient mixture F12 Ham (DMEM/F12, 1:1) supplemented with1 mM sodium pyruvate, 10% (v/v) fetal bovine serum (FBS), 100 Uml^{-1} penicillin, 100 μgml^{-1} streptomycin and 2.5 μgml^{-1} amphotericin B in a humidified atmosphere with 5% CO_2 at 37°C. All experiments were performed under the following protocol: cells grown to reach 85-95% confluency were harvested with trypsin-ethylenediaminetetraacetic acid and sub-cultured in culture dishes at a seeding density of $0.25x10^6$ cells per millimeter and continually cultured at 37°C in a humidified atmosphere with 5% CO_2.

In Vitro Cytotoxicity Assay

The cellular cytotoxicity of BP was tested on C6 glioma cells seeded into a 96-well plate ($1x10^4$ cells per well). After 26 h incubation, the cells were further incubated with fresh DMEM media (100 µl per well) containing BP in different concentrations of 0.09, 0.18, 0.375, 0.75, 1.5, 3.125, 6.25, 12.5, 25 and 50 µM for 24 and 48 h, respectively. Then the cells were washed with the culture media, and 100 µl of fresh DMEM containing 3-(4,5-dimethylthiazol-2-yl)-2,5-diphenyltetrazolium bromide (MTT-10 µl, 5 $mgml^{-1}$) was added to

each well followed by incubation for 4 h to allow the formation of formazan dye. The insoluble MTT formazan crystals were dissolved with DMSO (100 µl). The absorbance intensity at 570 nm was measured using a microplate reader (Synergy H1 hybrid multi-mode microplate reader, Bio-Tek). The relative cell viability (%) for each sample related to the control well was finally calculated.

Hippocampal Slice Preparation

For the preparation of hippocampal slices, standard procedure with minor modifications was adopted[1]. Adult male and female Sprague-Dawley rats weighing 250-300 g were anesthetized by isoflurane inhalation and decapitated. The brains were quickly removed and placed in ice-cold artificial cerebrospinal fluid (ACSF) containing (mM): NaCl 125, $NaHCO_3$ 25, KCl 2.5, NaH_2PO_4 1.25, $MgCl_2$ 1, D-glucose 10, $CaCl_2$ 2; saturated with 95% O_2 and 5% CO_2 at pH 7.4. The transverse slices of 400 µm thickness were prepared using Vibroslice NVSL with manually advanced tissue bath. All slice manipulation was done using phosphate free saline (PFS), because the presence of phosphate can precipitate zinc and thus limit the availability of free Zn^{2+} for detection. PFS was reported to cause no effect on transmission at the Schaffer collateral CA1 synapse suggesting that the neurons have substantial reserves of phosphate[2,3]. The PFS composition (mM) was: NaCl 125, $NaHCO_3$ 26, KCl 2.5, $MgSO_4$ 1.3, D-glucose 10, $CaCl_2$ 2; continuously bubbled with 95% O_2 and 5% CO_2, pH 7.4[3].

Zn^{2+} detection in acute hippocampal slices

Slices were separated into three groups and incubated with (i) PFS, (ii) PFS and 50 µM of CaEDTA, which is a classic extracellular zinc chelator, for 10-15 min and (iii) PFS and 50 µM of TPEN, which is an intracellular Zn^{2+} chelator, for 20 min at 37° C. For Zn^{2+} quantification, a minimum of three control and Zn^{2+} chelated slices were incubated with BP

and were imaged with a Rolera-XR Mono Fast 1394 Cooled digital camera (QImaging). Relative fluorescence intensity measured from three regions of interest for each slice was processed using ImageJ and quantified by the formula, CTCF=Integrated Density – (Area of selection x Mean fluorescence of background).

Quantification of Zn^{2+} in whole brain:

Adult Sprague-Dawley rats (>300 g) were euthanized and the brain was excised. Whole brain was weighed, incubated with BP probe (80 μM) and equally cut into 22 sections. Each section was weighed separately and imaged along with the solution having different concentration of zinc chloride incubated with BP probe in 96 well black plates. The quantification of free Zn^{2+} in whole brain of adult Sprague-Dawley rat was done by calculating the fluorescence pixel intensity of a particular section of brain assuming that the fluorescence from all brain sections are same. The pixel intensity of separate wells was measured by drawing ROI using living image software of IVIS. The ratio of pixel intensity of sample (different Zn^{2+} concentration) to pixel intensity of reference (BP alone) was plotted with different concentration of Zn^{2+}. From the average pixel intensity of brain section, the concentration of Zn^{2+} in particular section was calculated using calibration plot. By multiplying with the number of sections, the amount of Zn^{2+} in whole brain was calculated.

In vivo studies:

Establishing epileptic rat model

All animals used in this study were maintained under a 12/12h light/dark cycle at room temperature. Adult female Sprague-Dawley rats weighing 150 g were used as (1) control (n=3), injected with saline and (2) epilepsy model (n=6). Animals in the second group were given a single intra-peritoneal injection of pilocarpine hydrochloride (350mg/kg,

Sigma), freshly dissolved in 0.9% sterile saline and control rats received an injection of equal volume of sterile saline. The injection protocols were similar to those described previously[4,5]. To increase the survival rate, the pilocarpine treated animals received an intra-peritoneal injection of 0.5 mg/kg midazolam 1 h after the onset of status epilepticus (at stage IV and V) and when severe seizure was observed. Animals were observed under video-recording with iBall Face2Face C8 (Rev.3.0) camera for next 24 h for the signs of seizure activity and the seizures were classified according to Racine's scale (1972) and previous reports[6,7]: Normal activity of animal as stage 0, motionless stage for the first 10 min after pilocarpine injection as stage I, stiffened tail, head nodding and facial clonus as stage II, jerks in forelimb and whole body continuous clonic seizure as stage III, severe whole body continuous clonic seizure with rearing as stage IV and tonic-clonic seizure with falling or jumping due to loss of balance as stage V.

Intra-carotid artery infusion, Hyperosmolar BBB disruption and Zn^{2+} detection

The protocol was adapted from previous reports[8,9] with slight modification according to the needs. Control and epileptic rats were anesthetized by intra-peritoneal injection of ketamine @ 100mg/kg BW and xylazine @ 7mg/kg BW. After aseptic precautions, a paramedian longitudinal incision was made in the jugular furrow and left common carotid artery was exposed. The carotid artery was carefully separated out and 3-0 silk suture was passed underneath so as to lift the vessel and control bleeding. A sterile 26G cannula was then inserted into the lumen common carotid artery by direct puncture, with tip of the cannula directed towards the carotid bifurcation and was secured with silk sutures. Thereafter a ligature was placed proximal to the cannula. A small volume of heparinized PFS was flushed into the cannula to prevent blood coagulation and to confirm the forward flow towards the carotid bifurcation. The hub of the cannula was filled with PFS to minimize the introduction of air and 1.5 ml of PFS was infused through it for 1 min. PFS was aerated with 95% O_2-5%

CO_2, filtered through a 0.22 µm membrane filter (Millex-GP, no. SLGP033RS; Millipore Corp.) and warmed to 37°C prior to injection. To disturb the BBB, sterile 20% wt/vol mannitol (Mark Biosciences Ltd.) was warmed to 37°C and injected through the cannula into the external carotid artery for 30 s at a rate of 0.25 ml/kg/s. This dose has been reported to create a reversible BBB disruption without neuronal damage.

RESULTS

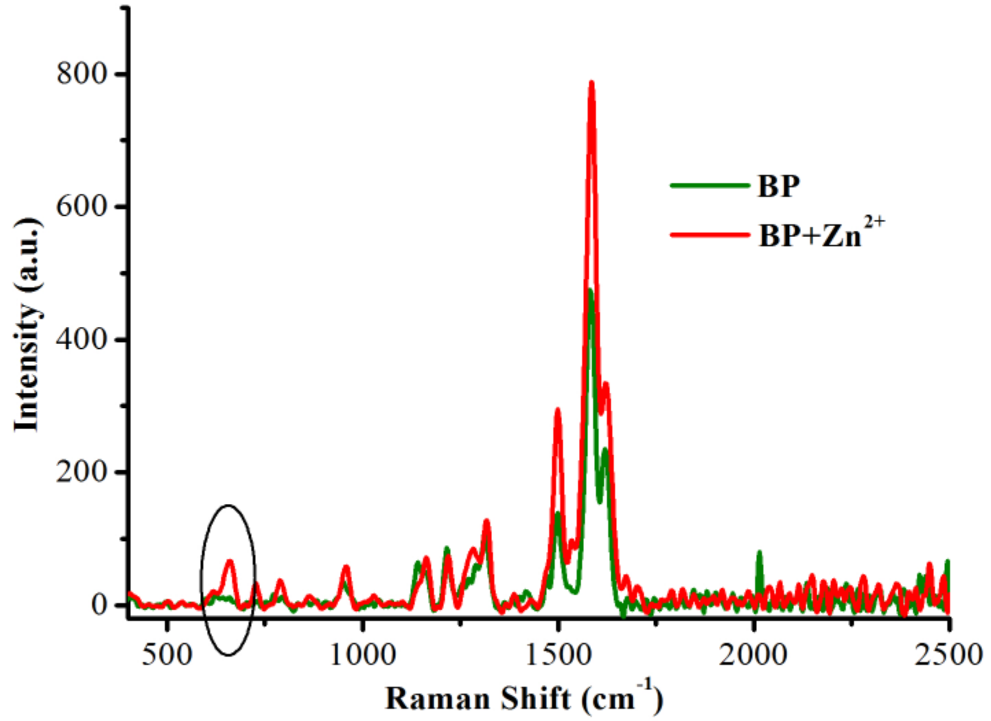

Figure S1: SERS spectra of BP with or without $ZnCl_2$

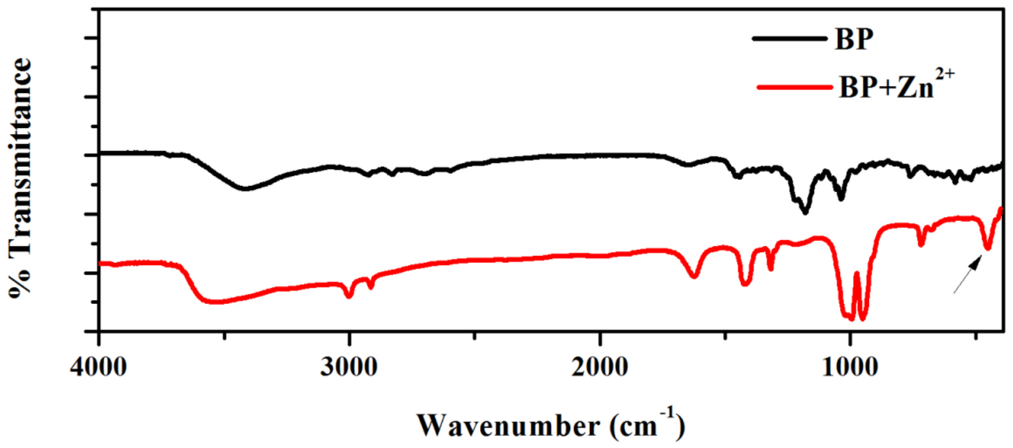

Figure S2: FTIR spectra of BP with or without $ZnCl_2$

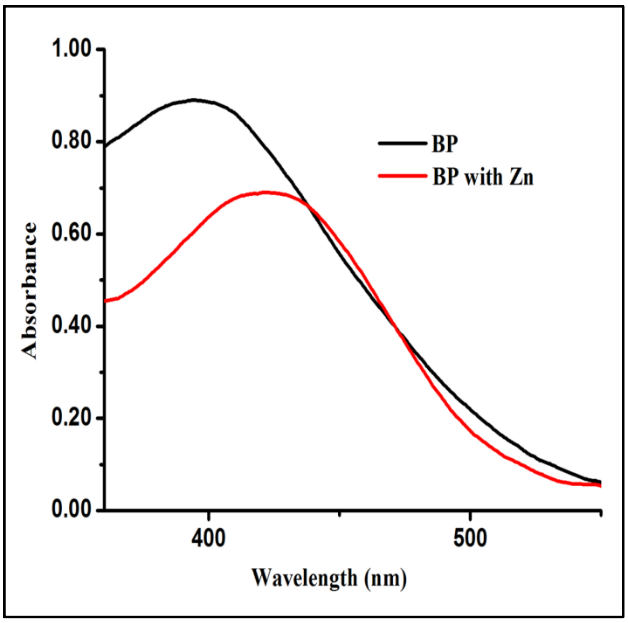

Figure S3: Absorption spectra of BP without or with $ZnCl_2$ in PFS (pH 7.4)

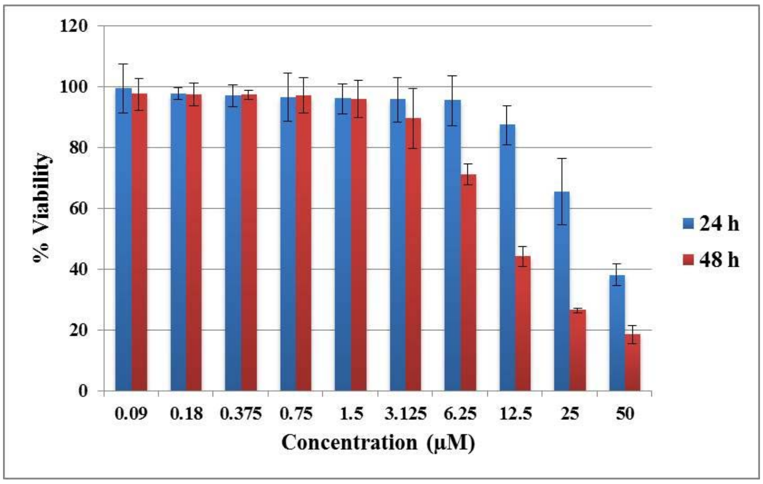

Figure S4: Cell viability (%) of C6 Glioma cells incubated with different concentrations (µM) of BP for 24 (blue) and 48 (red) h at 37°C. Error bar represents the Standard deviation(n=4).

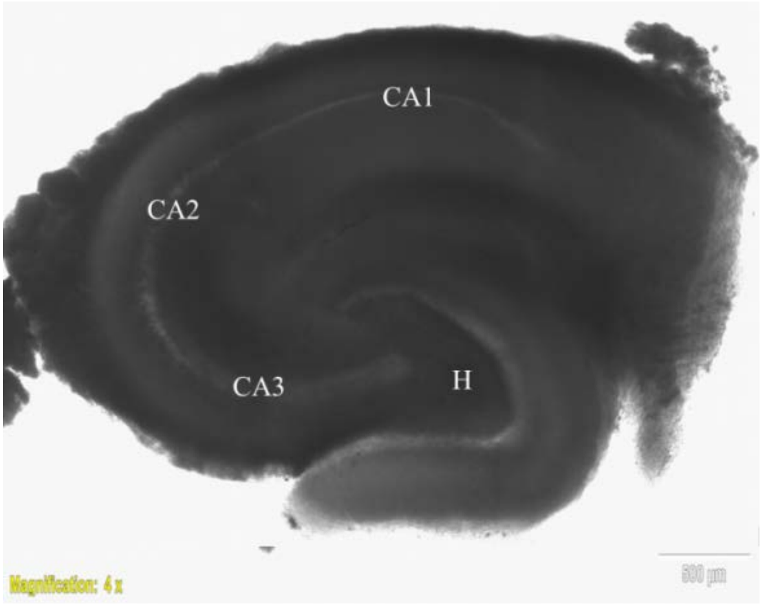

Figure S5: Microscopic image of 400 µM thick hippocampal slice sectioned perpendicular to the septo-temporal axis revealing the detailed lamellar anatomy. H marks the area of dentate hilus, CA1, 2 & 3 marks the cornuammonis 1, 2 & 3 respectively. Magnification 4X and scale bar = 500 µm.

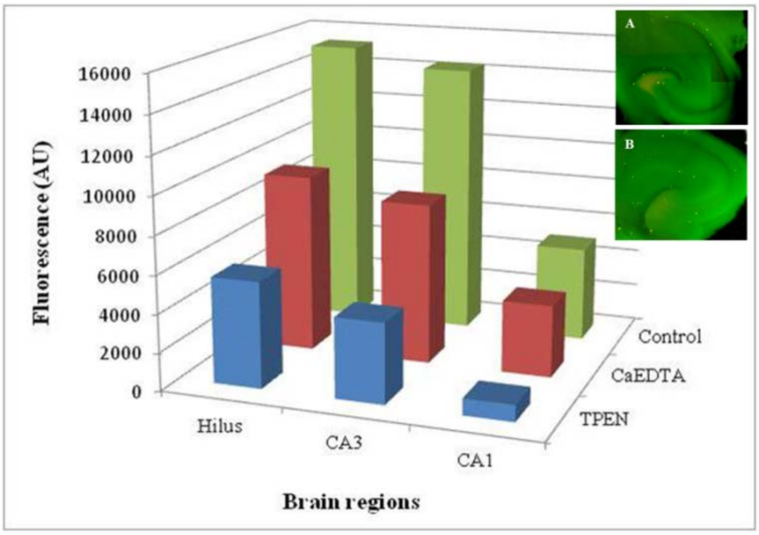

Figure S6: The effect of CaEDTA and TPEN on vesicular Zn^{2+}, imaged with BP. Hippocampal slices pre-treated with CaEDTA and TPEN were incubated with BP and

imaged. Relative fluorescence intensity from the three areas (hilus, CA3 & CA1) of hippocampus was measured and quantified. Inset shows the fluorescence images of BP incubated hippocampal slices pre-treated with CaEDTA (A) and TPEN (B) at 4X magnification.

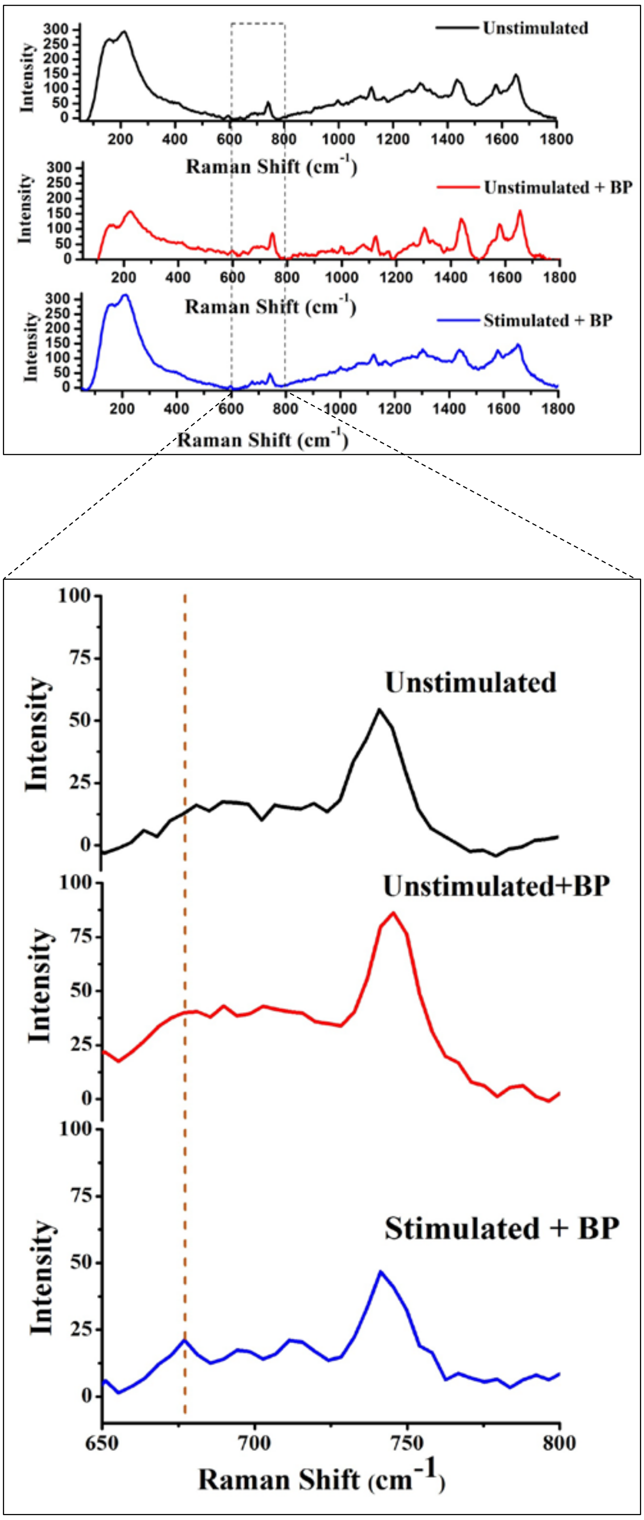

Figure S7: Raman Spectra from the hilar region of unstimulated (control) and stimulated (epileptic) hippocampal slices incubated with BP. Magnified spectrum shows the changes of characteristic vibrational peaks at 678 cm^{-1}.

Table S1: Raman peaks

**Raman Shift (cm^{-1})**				**Assignments**
**BP**	**Unstimulated Slice**	**Unstimulateds lice+BP**	**Stimulated slice+BP**	
	157-267	148-114	162-284	
	211-295	223-156	209-319	
	350-69	347-66	341-71	
	415-51	418-56	402-54	
		471-37		
	510-21	516-34		S-S Stretch
	542-12	545-29		
	563-8	572-25		
	596-11	599-30	597-6	Tyrosine
	637-3	640-23		
670-930				C-S stretch
		678-41	678-25	Metal-nitrogen bond
710-867	714-16		709-21	phospholipids such as phosphatidylethanolamine/ phosphatidylcholine
	741-56	741-88	740-46	Tryptophan
		821-16		
		848-18	863-29	Tyrosine
			889-33	
949-913		966-33	958-55	C-C of peptide backbone and BP, and PO_2^- stretching
	996-60	996-43	1001-70	Ring breathing-phenylalanine, PO_2^- stretch
1017-950	1022-51		1027-68	
			1057-83	
1070-962	1085-75	1079-51		Aliphatic side chain
	1120-102	1126-76	1122-111	Aliphatic side chain C-C stretch
	1165-75	1171-25	1161-91	
		1230-32	1230-102	
	1254-95	1263-55	1260-108	Amide III (alpha helix)& lipids

	1301-119	1304-102	1301-131	
	1340-97	1337-65		Tryptophan
	1358-83	1360-45	1352-102	
	1393-75	1390-38		
1415-1114	1435-131	1438-135	1438-130	CH_2deformation
			1494-75	
1529-1109		1541-49		
	1574-52	1577-114	1577-131	Trptophan
	1603-95		1609-112	Phenylalanine
	1654-147	1654-158	1652-148	Amide I alpha helix

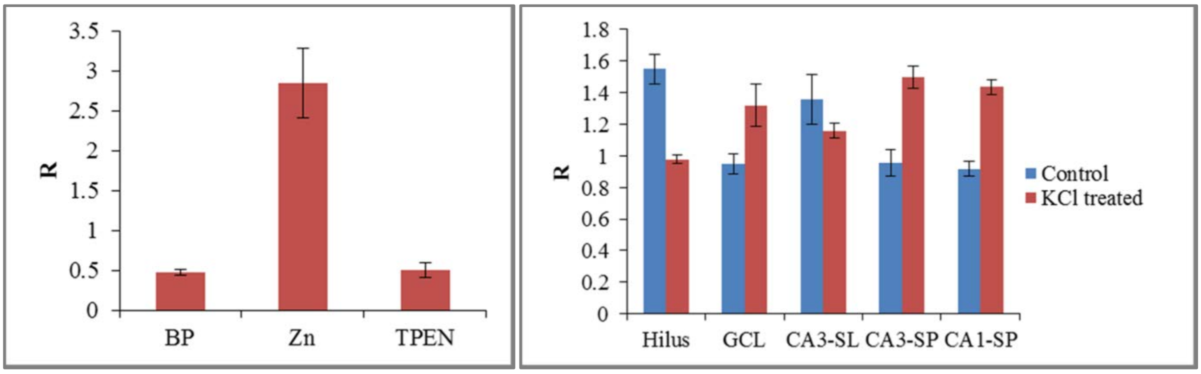

Figure S8: The average fluorescence emission intensity ratio ($R=I_{620}/I_{570}$) according to corresponding ratiometric images of cells in Fig. 5 (left) and slices in Fig. 6 (right). (n=5). Error bar represents the standard deviation.GCL-Granular cell layer of dentate gyrus, SL-Stratum lucidum, SP-Stratum pyramidale.

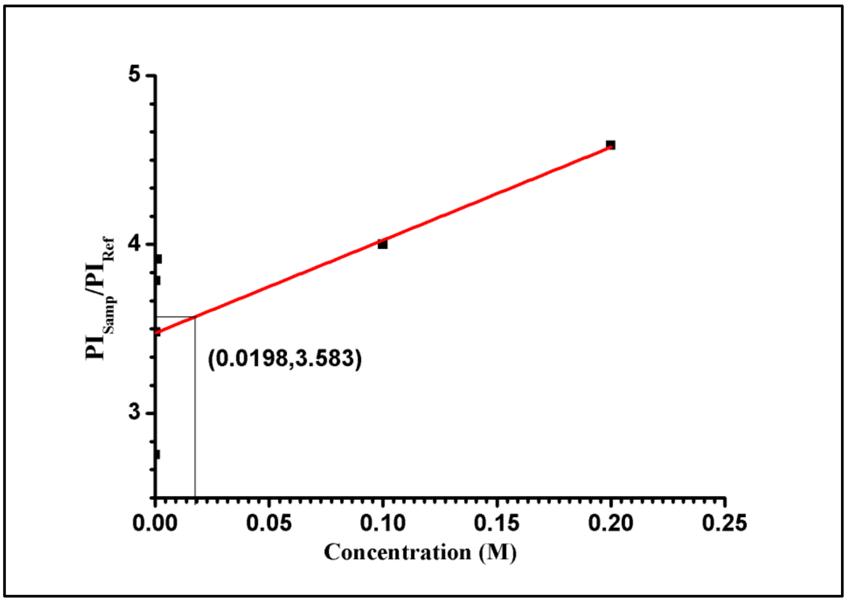

Figure S9: Quantification of total free Zn^{2+} in rat brain weighing 2.4 g. The calibration plot shows the pixel intensity with reference to different concentration of Zn^{2+}.

Behavioural changes observed during epilepsy induction:

All rats treated with pilocarpine showed reduced mobility for first 5-10 min. Intense salivation, mouth and facial movements (stage II) started 10 min after and progressed with head nodding, jerk in the forelimb for approximately 30 min. These stage III seizure become more intense after about 40 min, reflected in rearing and falling and eventually loss of balance (stage IV and V). After the injection of midazolam, an anticonvulsant[10,11],one of the rats underwent a slight sedation with decrease in seizure severity and retained the stage III seizure after 1 h, which was controlled with another injection of midazolam. Out of 6 pilocarpine treated rats, 4 rats exhibited status epilepticus (with stage IV/V seizures) which lasted for 3 to 4 h and the behavioural seizures end after 5-6 h and the animals survived. Epileptic animals were anesthetized after 24 h and taken for intra-carotid artery cannulation and BBB disruption for imaging.

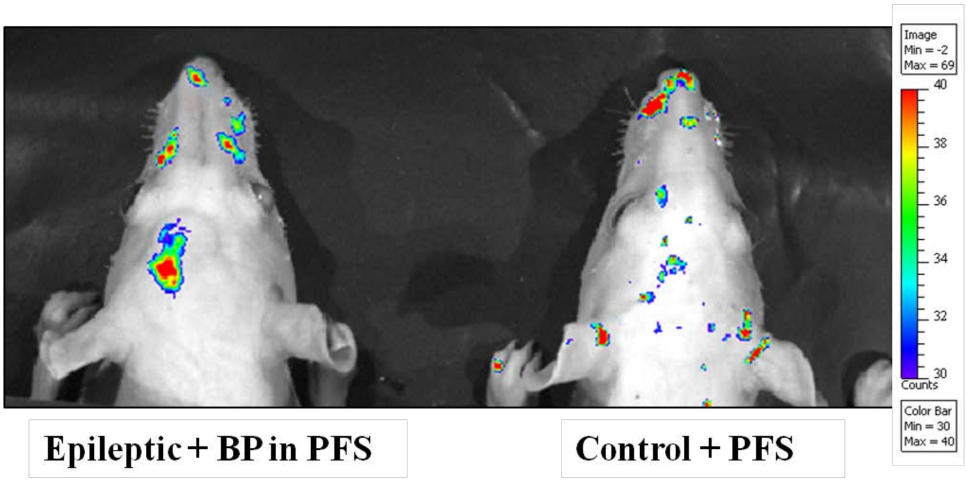

Figure S10: *In vivo* imaging of brain Zn^{2+} using BP at 430 nm excitation. Pilocarpine induced epileptic rat infused with BP (left) and control rat infused with PFS (right) are shown.

References:

1. Buskila, Y. *et al.* Extending the viability of acute brain slices. *Sci. Rep.* **4,** 5309 (2015).
2. Frederickson, C. J. *et al.* Synaptic release of zinc from brain slices: Factors governing release, imaging, and accurate calculation of concentration. *J. Neurosci. Methods* **154,** 19–29 (2006).
3. Rumschik, S. M., Nydegger, I., Zhao, J. & Kay, A. R. The interplay between inorganic phosphate and amino acids determines zinc solubility in brain slices. **108,** 1300–1308 (2009).
4. Long, J. J. A. *et al.* Pilocarpine model of temporal lobe epilepsy shows enhanced response to general anesthetics. *Exp. Neurol.* **219,** 308–318 (2009).
5. Houser, C. R. & Esclapez, M. Downregulation of the α5 subunit of the GABAA receptor in the pilocarpine model of temporal lobe epilepsy. *Hippocampus* **13,** 633–645 (2003).
6. Borges, K. *et al.* Neuronal and glial pathological changes during epileptogenesis in the mouse pilocarpine model. *Exp. Neurol.* **182,** 21–34 (2003).
7. Klitgaard, H., Matagne, A., Grimee, R., Vanneste-Goemaere, J. & Margineanu, D. G. Electrophysiological, neurochemical and regional effects of levetiracetam in the rat pilocarpine model of temporal lobe epilepsy. *Seizure* **12,** 92–100 (2003).
8. Chertok, B., David, A. E. & Yang, V. C. Polyethyleneimine-modified iron oxide nanoparticles for brain tumor drug delivery using magnetic targeting and intra-carotid administration. *Biomaterials* **31,** 6317–6324 (2010).
9. Foley, C. P. *et al.* Intra-arterial delivery of AAV vectors to the mouse brain after mannitol mediated blood brain barrier disruption. *J. Control. Release* **196,** 71–78 (2014).
10. McMullan, J., Sasson, C., Pancioli, A. & Silbergleit, R. Midazolam versus Diazepam for the treatment of status Epilepticus in children and young adults: A meta-analysis. *Acad. Emerg. Med.* **17,** 575–582 (2010).
11. Reddy, S. D. & Reddy, D. S. Midazolam as an anticonvulsant antidote for organophosphate intoxication - A pharmacotherapeutic appraisal. *Epilepsia* **56,** 813–821 (2015).
